# Low serum 25‐hydroxyvitamin D3 levels and late delayed radiation‐induced brain injury in patients with nasopharyngeal carcinoma: A case–control study

**DOI:** 10.1002/brb3.1892

**Published:** 2020-10-25

**Authors:** Zhezhi Deng, Minping Li, Junjie Guo, Dongxiao Zhou, Xurui Huang, Yongxin Huang, Haiwei Huang

**Affiliations:** ^1^ Department of Neurology The First Affiliated Hospital Sun Yat‐sen University Guangdong Provincial Key Laboratory of Diagnosis and Treatment of Major Neurological Diseases National Key Clinical Department and Key Discipline of Neurology Guangzhou China

**Keywords:** 25‐hydroxyvitamin D3, biomarker, immune inflammatory effects, radiation‐induced brain injury

## Abstract

**Background and Purpose:**

Inflammatory reaction plays a critical role in the pathogenesis of late delayed radiation‐induced brain injury (RBI). Low vitamin D levels are closely associated with various immuno‐inflammatory diseases, but the relationship with late delayed RBI remains unknown. Here, we aimed to determine the association of serum vitamin D levels with clinical parameters in late delayed RBI patients with nasopharyngeal carcinoma.

**Methods:**

25‐Hydroxyvitamin D3 levels and clinical and cerebrospinal fluid parameters were evaluated in 21 patients with RBI and compared with 90 age‐, sex‐, and season‐matched healthy controls.

**Results:**

25‐(OH)D3 levels were lower in patients with RBI compared to controls (40.39 ± 22.11 vs. 64.54 ± 19.89 nmol/L, *p* < .001), especially for aged ≥60 years (vs. <60 years, *p* = .038), females (vs. males, *p* = .012), short latency (<5 years) (vs. ≥5 years, *p* = .015), and severe impairment (LENT/SOMA score ≥3) (vs. LENT/SOMA score <3, *p* = .010). Serum 25‐(OH)D3 levels were associated with age (*r* = −.464, *p* = .015), Latency of RBI (*r* = .416, *p* = **.031**) and LENT/SOMA Scale (*r* = −.488, *p* = .010).

**Conclusions:**

Our data showed that serum 25‐(OH)D3 levels were reduced in late delayed RBI patients with nasopharyngeal carcinoma.

## Graphical abstract

Inflammatory reaction plays a critical role in the pathogenesis of RBI. Low vitamin D levels are closely associated with various immuno‐inflammatory diseases, but the relationship with late delayed RBI remains unknown. Here, we reported that patients with RBI had significantly lower serum levels of 25(OH)D3 than age‐, season‐ and sex‐matched control subjects. Furthermore, low levels of 25(OH)D3 were associated with female sex, older age, short latency, and disease disability.

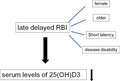

## 1 INTRODUCTION

Radiation‐induced brain injury (RBI) is the most serious complication after radiation therapy for head and neck tumors such as nasopharyngeal carcinoma (NPC), which can be divided into three types based on classic radiobiological perspectives and timeline: acute (several days to several weeks); sub‐acute or early delayed (12 weeks to 6 months); and late delayed (>6 months) (Brandsma et al., 2008). Late delayed RBI has the highest incidence and the most severe symptoms, can even be life‐threatening, and often shows progressive intracranial pressure caused by extensive cerebral edema, cognitive dysfunction, and other neurological deficit (Schultheiss et al., 1995). Immune inflammatory processes play a vital role in the development of RBI, so immunosuppressant such as corticosteroids are commonly used in clinical treatment (Balentova & Adamkov, 2015).

Vitamin D is an organic compound necessary for modulating metabolism of calcium and phosphorus, which is mainly synthesized from 7‐dehydrocholesterol in the skin by the action of ultra violet light. Increasing studies have found that vitamin D is a selective immunomodulator in addition to traditional physiological functions (Sassi et al., 2018). When the body is in an immunosuppressive state, vitamin D enhances the function of monocytes and macrophages. Conversely, when the immune function abnormally increases, vitamin D inhibits the proliferation of activated T and B lymphocytes to maintain immune balance. It is worth noting that vitamin D itself has no physiological function, and only when it is converted into its active form can it become a physiologically active substance. The major active form in circulation is 25‐hydroxyvitamin D3 (25‐(OH)D3), which can be measured to assess vitamin D status (Holick, 2007).

Recent studies confirmed that low levels of serum 25‐(OH)D3 are associated with a variety of immune disorders such as systemic lupus erythematosus (SLE), rheumatoid arthritis (RA), and autoimmune thyroid diseases (Harrison et al., 2020; Islam et al., 2019; Lee et al., 2019). Moreover, vitamin D is independent risk factor for CNS demyelination including multiple sclerosis (MS), neuromyelitis optica spectrum disorder (NMOSD), and inflammatory spinal cord disease (Mealy et al., 2012; Salzer et al., 2012; Shan et al., 2016). In addition, studies have shown that serum 25‐(OH)D3 levels were reduced in patients with anti‐N‐methyl‐d‐aspartate receptor encephalitis (Shu et al., 2017). Furthermore, studies have pointed out Vitamin D deficiency is associated with the severity of radiation‐induced proctitis in cancer patients (Ghorbanzadeh‐Moghaddam et al., 2015). However, the relationship between low serum 25‐(OH)D3 levels and RBI remains unknown. Here, we analyzed 25‐(OH)D3 levels in late delayed RBI patients and determined the association of vitamin D levels with clinical parameters in these patients.

## 2 METHODS

### 2.1 General information

This was a single‐center cross‐sectional study based on the patients of the First Affiliated Hospital of Sun Yat‐sen University, which is approved by the medical ethics committee of the First Affiliated Hospital of Sun Yat‐sen University.

From 1 October 2018 to 31 December 2019, we recruited consecutive patients with late delayed RBI (*n* = 27), and age‐, sex‐, and season‐matched healthy controls (CTLs, *n* = 80) were selected from the medical Examination Center of First Affiliated Hospital of Sun Yat‐sen University. The irradiation dose was 67–75 Gy with a fraction dose of 2 Gy. Season was defined as spring (March to May), summer (June to August), fall (September to November), and winter (December to February) and was also acquired and matched to healthy controls. Inclusion criteria included: (a) age ≥18 years old; (b) Radiation therapy history for histologically confirmed NPC > 6 months prior to study entry; (c) Radiographic evidence to support the diagnosis of RBI without tumor recurrence; (d) No history of glucocorticoid use in the last 3 months; (e) Signed an informed consent form. Patients were excluded if with other intracranial inflammatory or infectious diseases.

Magnetic resonance imaging (MRI) and CSF examinations were reviewed. Neurological signs and symptoms were assessed by the Late Effects of Normal Tissue (LENT)/Subjective, Objective, Management, Analytic (SOMA) scales (Routledge et al., 2003). The LENT/SOMA scale contains 4 domains. Each domain scores from 1 to 4, with a 0 score if there are no toxicities. We defined the final grade of the LENT/SOMA scale as the maximum score of the 4 components. Symptoms were categorized into the following six groups: (a) Cranial nerve palsy such as dysarthria and dysphagia; (b) Cranial hypertension symptoms such as headache, nausea, and vomiting; (c) Cognitive impairment symptoms such as memory deficits and mental disorders; (d) Conduction beam damage such as limb weakness and numbness; (e) Cerebellar disorder such as vertigo and ataxia; (f) Sleep disorder.

### 2.2 Vitamin D measurements

Serum 25‐(OH)D3 levels were measured with a commercially available enzyme‐linked immunosorbent assay kit (Immunodiagnostic Systems Limited, Cat#: AC‐57SF1, RRID: AB_2756867), according to the manufacturer's instructions. Levels of 25‐(OH) D3 < 50 nmol/L were determined to be deficient and 50 nmol/L but <75 nmol/L as insufficient (Holick, 2007).

### 2.3 MRI scanning

**FIGURE 1:**
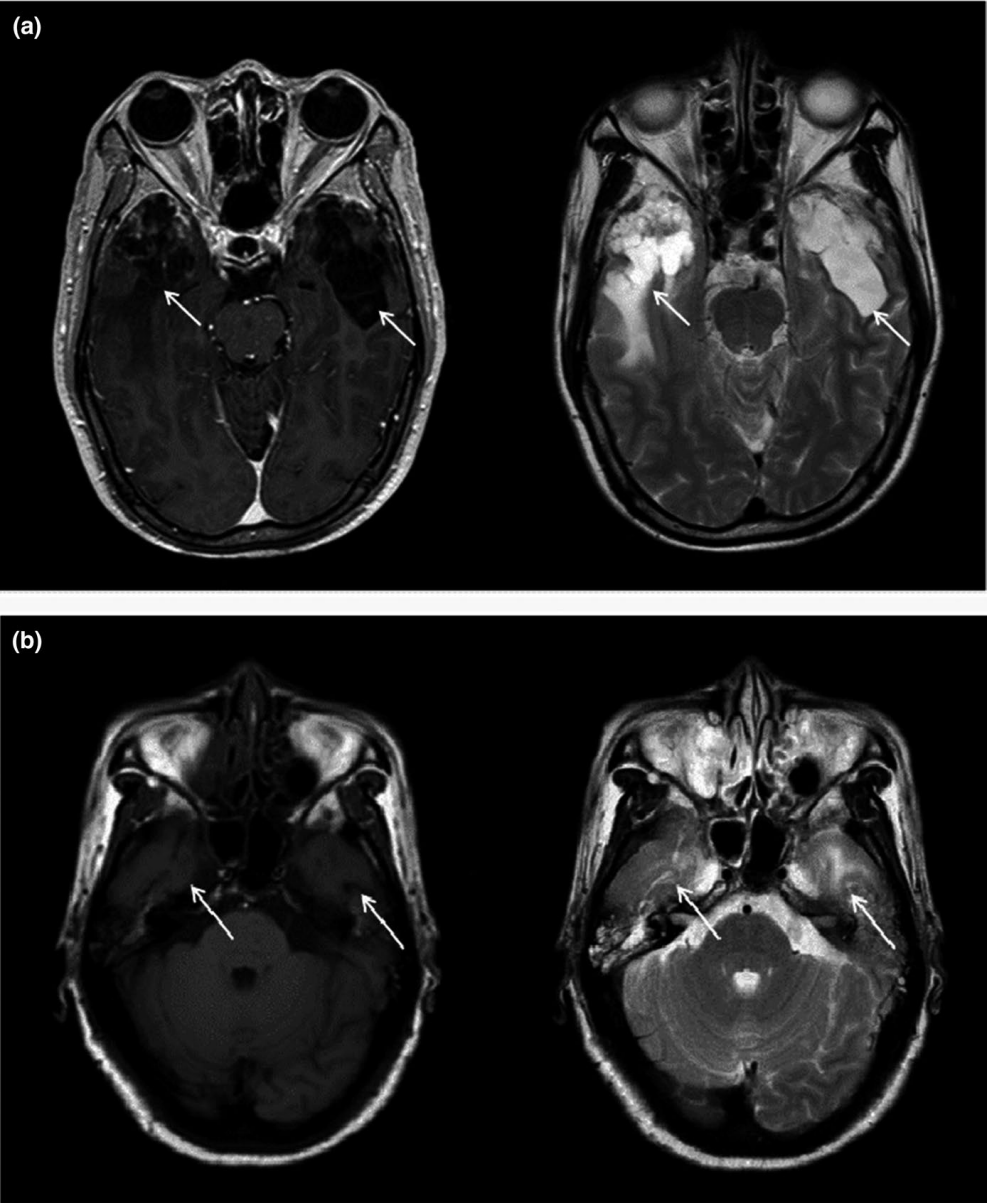
Representative MRI images of a patient with RBI. (a) T2‐weighted image showing finger‐like edema with bilateral lesions in temporal lobe and gadolinium‐enhanced T1‐weighted image show spot‐like and strip‐like enhancements with the right side. (b) T2‐weighted image showing small patchy abnormal signal shadows in bilateral temporal lobes and T1‐weighted image of the same patient

Cranial MRI scanning was used for RBI patients by a GE 1.5T MR scanner (General Electric). The slice thickness of axial scans was 5 mm. Gadopentetate dimeglumine (Gd‐DTPA) was administered intravenously for enhanced scanning at a dose of 0.1 mmol/kg. Figure 1 illustrates representative images of the T2‐weighted and heterogeneous contrast enhancement in gadolinium‐enhanced T1‐weighted images of 2 patients with RBI.

### 2.4 Statistical analysis

All the data were analyzed by SPSS 22.0. Measurement data were expressed as mean ± standard deviation (*SD*) and were compared with Student's *t* test for normally distributed variables. Non‐normal distribution measurement data were described using the median and interquartile range and were compared using Mann–Whitney *U* test. Categorical data were given as the numbers and percentage (%) and were compared by chi‐square test and Fisher's exact test. Correlations between serum 25‐(OH)D3 and age, BMI, LENT/SOMA score, and CSF factors (CSF WBC, TP, Glu, and CL) were analyzed by Spearman's rank test. All analyzes were two‐sided, and *p* < .05 for the difference was statistically significant.

## 3 RESULTS

### 3.1 General characteristics of RBI patients and healthy controls

The details of general characteristic including demographics, clinical features, and laboratory data between RBI patients and healthy controls are summarized in Table 1. A total of 27 RBI patients (mean age, 54.56 ± 11.34 years; mean BMI, 22.26 ± 2.95; male:female = 16:11; spring:summer:fall:winter = 6:3:10:8), and 80 CTLs (mean age, 53.64 ± 10.31 years; mean BMI, 21.71 ± 3.30; male:female = 48:32; spring:summer:fall:winter = 17:10:28:25) were included in our study. The median LENT/SOMA score in RBI patients was 2 (range, 1–4). All primary tumors of RBI patients are NPC. Of 27 patients with RBI, 5 patients (18.5%) had cranial nerve palsy such as dysarthria and dysphagia; 17 patients (63.0%) had cranial hypertension symptoms such as headache, nausea, and vomiting; 20 patients (74.1%) had cognitive impairment symptoms such as memory deficits and mental disorders; 9 patients (33.3%) had conduction beam damage such as limb weakness and numbness; 6 patients (22.2%) had cerebellar disorder such as vertigo and ataxia; 18 patients (66.7%) had sleep disorder. In addition, as shown in Table 1, there is no statistical difference in the underlying disease rates between the two groups.

**TABLE 1:** Demographic features of patients with RBI and healthy controls

	RBI (*n* = 27)	CTLs (*n* = 80)	*p*
Age (year)	54.56 ± 11.34	53.64 ± 10.31	ns
Gender (*n*, %)			
Male	16 (59.3%)	48 (60.0%)	ns
Female	11 (40.7%)	32 (40.0%)	ns
BMI	22.26 ± 2.95	21.71 ± 3.30	ns
Season (*n*, %)			
Spring	6 (22.2%)	17 (21.3%)	ns
Summer	3 (11.1%)	10 (12.5%)	ns
Fall	10 (37.0%)	28 (35.0%)	ns
Winter	8 (29.7%)	25 (31.2%)	ns
Primary tumor	NPC	–	–
Latency of RBI (year)	6.1 ± 2.9	–	–
LENT/SOMA scale (*n*, %)		–	–
Grade 1 (*n*, %)	3 (29.7%)	–	–
Grade 2 (*n*, %)	12 (29.7%)	–	–
Grade 3 (*n*, %)	10 (29.7%)	–	–
Grade 4 (*n*, %)	2 (29.7%)	–	–
Lesion sites (*n*, %)			
Temporal lobe	23 (85.2%)	–	–
Other	4 (14.8%)	–	–
Symptoms (*n*, %)			–
Cranial nerve palsy	5 (18.5%)	–	–
Cranial hypertension symptoms	17 (63.0%)	–	–
Cognitive impairment symptoms	20 (74.1%)	–	–
Conduction beam damage (limb weakness and numbness)	9 (33.3%)	–	–
Cerebellar injury symptoms	6 (22.2%)	–	–
Sleep disorder	18 (66.7%)	–	–
25(OH)D3 level (nmol/L)	40.39 ± 22.11	64.54 ± 19.89	**<.001**
25(OH)D3 level <50 nmol/L (*n*, %)	17 (63.0%)	18 (22.5%)	
25(OH)D3 level ≥50, <75 nmol/L (*n*, %)	8 (29.6%)	40 (50.0%)	
25(OH)D3 level ≥75 nmol/L (*n*, %)	2 (7.4%)	22 (27.5%)	
CSF routine			
CSF WBC (×10^6^, median, range)	5.0 (1.0–8.0)	–	–
CSF TP (g/L, mean ± *SD*)	0.42 ± 0.15	–	–
CSF Glu (mmol/L, mean ± *SD*)	3.78 ± 0.74	–	–
CSF CL (mmol/L, mean ± *SD*)	122.0 ± 1.41	–	–
WBC (×10^9^, median, range)	4.9 (2.4–5.8)	6.3 (3.7–8.8)	ns
Underlying disease			
Coronary heart disease (*n*, %)	0 (0.0%)	1 (1.2%)	ns
Hypertension (*n*, %)	10 (37.0%)	27 (33.8%)	ns
Diabetes mellitus (*n*, %)	7 (25.9%)	20 (25.0%)	ns

### 3.2 Comparison of serum 25(OH)D3 levels between patients with RBI and healthy controls

The mean concentration of serum 25‐(OH)D3 in patients with RBI was 40.39 ± 22.11 nmol/L compared with 64.54 ± 19.89 nmol/L in CTLs, (*p* < .001, Table 1). Among the 27 patients with RBI, 17 (63.0%) showed vitamin D deficiency (<50 nmol/L), 8 patients (29.6%) had vitamin D insufficiency (50–75 nmol/L), and only 2 patients (7.4%) had a sufficient vitamin D level (75 nmol/L). By contrast, 18 (22.5%) of 90 CTLs were considered vitamin D deficient, 40 (50.0% had insufficient levels, and 22 (27.5%) having sufficient levels (Table 1).

### 3.3 Comparison of serum 25‐(OH)D3 levels between subgroups of patients with RBI

For further study, we subdivided patients with RBI into two subgroups by gender, age, BMI, Season, LENT/SOMA scale, Latency of RBI, Lesion sites, with or without specified symptoms, and underlying disease (Table 2). The serum 25‐(OH)D3 level in male patients was significantly higher than in female patients (*p* = .012). Older patients (age ≥60 years) had lower serum 25‐(OH)D3 levels than those age <60 years (*p* = .038). Patients with latency of RBI < 5 had lower serum 25‐(OH)D3 levels than those with latency of RBI ≥ 5 (*p* = .015). Serum 25‐(OH)D3 levels in patients with latency of LENT/SOMA scale <3 were significantly higher than in patients with LENT/SOMA scale ≥3 (*p* = .010). Lesion site has no significant effect on serum 25‐(OH)D3 levels (*p* = .688). Although patients with conduction beam damage had significantly lower serum 25‐(OH)D3 levels than those without conduction beam damage (*p* = .003), no significant difference in serum 25‐(OH)D3 levels between patients with other symptoms and those without. In addition, patients with underlying disease had lower serum 25‐(OH)D3 levels than those without. (*p* = .017) (Table 2).

**TABLE 2:** 25‐(OH)D3 levels in patients with RBI

Variables	Mean ± *SD* (nmol/L)	Range (nmol/L)	*p*
Age (years)			
<60 (*n* = 17)	47.08 ± 20.14	12.75–76.23	
≥60 (*n* = 10)	29.02 ± 6.80	7.56–63.13	**.038**
Gender			
Male (*n* = 16)	48.90 ± 5.22	11.46–76.23	
Female (*n* = 11)	28.01 ± 5.50	7.56–63.13	**.012**
BMI			
<20 (*n* = 7)	40.77 ± 9.19	7.56–75.72	
≥20 (*n* = 20)	40.26 ± 4.91	11.46–76.23	.959
Season (*n*, %)			
Spring (*n* = 6)	31.51 ± 24.97	7.56–66.76	
Summer (*n* = 3)	43.86 ± 32.27	11.69–76.23	
Fall (*n* = 10)	42.09 ± 18.84	12.87–69.44	
Winter (*n* = 8)	43.62 ± 22.97	11.46–75.72	.428
Latency of RBI (year)			
<5 (*n* = 10)	27.30 ± 6.08	7.56–76.23	
≥5 (*n* = 17)	48.09 ± 4.95	11.46–66.76	**.015**
LENT/SOMA scale			
Grade 1–2 (*n* = 15)	49.88 ± 22.43	11.46–52.88	
Grade 3–4 (*n* = 12)	28.53 ± 15.46	7.56–76.23	**.010**
Lesion sites			
Temporal lobe (*n* = 24)	41.12 ± 4.51	7.56–76.23	
Other (*n* = 4)	36.16 ± 13.92	12.87–75.72	.688
Symptoms			
Cranial nerve palsy			
With (*n* = 6)	46.66 ± 6.19	26.63–69.44	
Without (*n* = 21)	38.60 ± 5.18	7.56–76.23	.442
Cranial hypertension symptoms			
With (*n* = 17)	37.14 ± 4.86	7.56–69.44	
Without (*n* = 10)	45.91 ± 8.03	11.46–76.23	.329
Cognitive impairment symptoms			
With (*n* = 20)	36.69 ± 4.65	11.46–76.23	
Without (*n* = 7)	50.96 ± 9.04	7.56–69.44	.145
Conduction beam damage (limb weakness and numbness)			
With (*n* = 9)	23.39 ± 5.94	7.56–61.77	
Without (*n* = 18)	48.89 ± 4.53	11.46–76.23	**.003**
Cerebellar injury symptoms			
With (*n* = 6)	34.81 ± 9.50	11.69–63.13	
Without (*n* = 21)	41.99 ± 4.82	7.56–76.23	.494
Sleep disorder			
With (*n* = 17)	39.12 ± 5.82	7.56–76.23	
Without (*n* = 10)	42.55 ± 6.17	14.45–69.08	.705
Underlying disease			
With (*n* = 11)	28.45 ± 5.61	7.56–63.13	
Without (*n* = 16)	48.60 ± 5.25	11.46–76.23	**.017**

### 3.4 Correlation between serum 25‐(OH)D3 levels, clinical characteristics, and CSF parameters in RBI patients

The relationship between serum 25‐(OH)D3 levels, clinical characteristics, WBC, and CSF parameters in RBI patients was evaluated (Table 3). It was shown that there was a negative correlation between serum 25‐(OH)D3 levels and age (*r* = −.464, *p* = .015). Furthermore, there was a positive correlation between serum 25‐(OH)D3 levels and latency (*r* = .416, *p* = .031). In contrast, there was a negative correlation between serum 25‐(OH)D3 levels and LENT/SOMA score. (*r* = .488, *p* = .010). However, correlations between serum 25‐(OH)D3 levels and BMI, and WBC and CSF parameters (CSF WBC, CSF TP, CSF Glu, CSF CL) were not significant (Table 3).

**TABLE 3:** Correlation coefficients generated between serum 25‐(OH)D3 levels and clinical characteristics, CSF parameters in RBI patients

Variables	*r*_s_	*p*
Age	−.464	**.015**
BMI	.050	.806
Latency of RBI	.416	**.031**
LENT/SOMA scale	−.488	**.010**
CSF WBC	−.472	.422
CSF TP	.059	.926
CSF Glu	−.251	.684
CSF CL	−.340	.576
WBC	.038	.936

## 4 DISCUSSION

Emerging evidences have shown that vitamin D not only plays a physiological role in calcium metabolism and bone growth, but also has a strong immune‐regulating function. Interestingly, diffuse T‐cell infiltration and activated infiltrated macrophages with high expression of proinflammatory factors can be seen in the frozen sections of brain biopsies from RBI patients (Kureshi et al., 1994). Normally, the presence of the blood–brain barrier prevents peripheral immune cells from entering the central nervous system. In our previous studies, it was found that ionizing radiation can induce a decrease in tight junction protein expression (Deng et al., 2017). Increased vascular permeability and disruption of the blood–brain barrier after irradiation can lead peripherally derived macrophages and white blood cells to enter the central nervous system.

Increasing research indicates that vitamin D levels are closely related to radiation‐induced inflammatory damage (Yazici et al., 2011). However, there is poorly no report about the relationship between vitamin D in RBI. This study analyzed the active form of vitamin D (25‐(OH)D3) levels in the serum of patients with late delayed RBI. It was shown that patients with RBI had significantly lower levels of 25‐(OH)D3 than age‐, season‐, and sex‐matched controls. Moreover, low levels of 25‐(OH)D3 are closely related to female, older age, the presence of underlying disease, shorter incubation period, and worse prognosis of the disease.

The effect of vitamin D in anti‐inflammatory and immunomodulatory may be the link to RBI. Vitamin D has extensive physiological effects in regulation of innate and adaptive immunity, which is mediated by signaling through the vitamin D receptor expressed on immune cells (Delvin et al., 2014). It was reported that vitamin D regulates the proliferation and transformation of monocyte and macrophages (Yuk et al., 2009). In addition, vitamin D controls T‐cell antigen receptor signaling and activation of human T cells, while inhibits the proliferation of activated B cells and induced their apoptosis (Chen et al., 2007). In human innate lymphoid cells, Vitamin D downregulates the IL‐23 receptor pathway (Konya et al., 2018). Clinical studies have shown associations of vitamin D deficiency with various immune diseases such as SLE, RA, and inflammatory bowel disease (IBD) (Agmon‐Levin et al., 2013; Bae & Lee, 2018; Kabbani et al., 2016). In central nervous system (CNS), adaptive immunity is the key to the understanding of autoimmune and paraneoplastic inflammatory central nervous system disorders such as MS, NMOSD, and autoimmune encephalitis, which have also been shown to be closely related to vitamin D deficiency (Weissert, 2017). Disturbance of autoimmune system caused by ionizing radiation promotes the development of RBI. Consistent with other neurological autoimmune diseases, we found that serum vitamin D levels were significantly reduced in RBI patients in this study. Recently, cumulative evidence links vitamin D and radiation‐induced injury. Therefore, we speculate that low serum vitamin D may be a risk factor for developing RBI, especially late delayed RBI, as it might affect immune cells. More extensive evidences of vitamin D in the pathogenesis of RBI are required.

In our study, lower concentrations of serum 25‐(OH)D3 were associated with short latency and high LENT/SOMA scores in RBI, which maybe means that vitamin D deficiency accelerates the development and increases the incidence of disease. In accord with the previous study, we also found that female patients with RBI had lower vitamin D levels compared with male patients. These findings might be less calcium loss in men compared with women. In addition, due to the unique esthetics of Asian women, they are more willing to perform indoor activities to prevent sun exposure and tanning, which may also be a potential cause. Vitamin D is mainly derived from sunlight, which has a clear seasonality. However, our results showed no clear seasonal differences. This may be because Guangdong is located in the tropical‐subtropical zone, and there is no obvious difference in sunshine in the four seasons. Elderly patients are often accompanied by underlying diseases. In the elderly patients with limb weakness and seriously ill patients, it may be due to limited exercise and lack of outdoor sports, resulting in insufficient sunlight.

This study had several limitations. First, controlled experiments with NPC patients without radiation damage are missing. Second, the sample size of RBI patients was small. Third, we did not investigate the role of immune cells such as T or B cells and vitamin D receptor expression in relation to the mechanisms underlying disease pathogenesis of RBI.

In conclusion, we report that serum 25‐(OH)D3 levels are reduced in late delayed RBI patients and are associated with disease disability.

## CONFLICT OF INTEREST

The authors declare no potential conflict of interest.

## AUTHOR CONTRIBUTION

Zhezhi Deng and Haiwei Huang designed this research; Minping Li, Junjie Guo, Dongxiao Zhou, Xurui Huang, and Yongxin Huang performed data collection; Zhezhi Deng and Minping Li performed data analysis; Zhezhi Deng and Haiwei Huang wrote the manuscript; all authors read and approved the final manuscript.

## ETHICAL APPROVAL

This study was approved by the medical ethics committee of the First Affiliated Hospital of Sun Yat‐sen University.

### Peer Review

The peer review history for this article is available at https://publons.com/publon/10.1002/brb3.1892.

## Data Availability

The data that support the findings of this study are available from the corresponding author upon reasonable request.
